# The use of a simple 8 bit microprocessor as a flexible sequence controller for developing laboratory automation

**DOI:** 10.1155/S1463924680000065

**Published:** 1980

**Authors:** F. Morley

**Affiliations:** Laboratory of the Government Chemist Cornwall House Stamford Street London SE1 9NQ UK


### The use of a simple 8 bit microprocessor as a flexible sequence controller for developing laboratory automation

F. Morley
*Laboratory of the Government Chemist, Cornwall House, Stamford Street, London SE1 9NQ, UK.*

### Introduction

The Laboratory of the Government Chemist carries out a number of different routine chemical analyses for its customers. In order to carry out these analyses in the most efficient and cost effective way many of the analytical procedures have been automated, [1, 2]. Automatic data capture for analysis by computer is also widely used, [3]. The development of automatic systems used in the Laboratory is carried out by a small team of specialists with backgrounds in instrumentation, analytical chemistry and electronics. Some typical examples of systems developed by the team include the automatic determination of metals in food, [4], the determination of density of alcohol, [5], and the automatic dilution and measurement of samples for vitamin assays. One of the difficulties encountered in constructing this type of system is the control of the many valves, turntables, dispensers and similar types of devices, typically 10 – 20 per system. In earlier systems developed prior to microprocessors the control was achieved using cam timers which worked on a fixed time cycle, each cam being set at the correct time point for the component it controlled. In addition to being outmoded, this method also suffers from severe problems whenever one element has to be changed, since a change in one element will effect all those following, and these then also have to be reset.

The next stage in the control of systems was achieved using solid state timers. Whilst such devices provide a greater flexibility when altering individual elements, they still suffer from the problem of cascading the change to all the following elements. This method is also quite costly as a number of different timing modules have to be available for making changes. The third generation of control is in the form of sequential logic controllers built from transistor logic circuits (TTL). As well as being a time based operation, this mode of control enables event initiated operation far more easily than the earlier systems. It also has the advantage of requiring relatively cheap circuitry. The disadvantages of this type of system are that it is less easy to change than discrete timers and the logic circuits themselves have to be buffered by other devices, eg. relays, to allow them to drive the system components.The introduction of inexpensive microprocessor technology allows considerable improvement in the flexibility of the system control and the programmable controller becomes a reality. The major advantage with a microprocessor system is flexibility. This applies not only to the changing of parameters but also to redesigning the way in which the control system functions. It is quite feasible to use the same controller for a wide variety of applications with only a simple program change. The microprocessor system thus offers several advantages to a development section where many changes may have to be made during the development of an automatic instrument. This paper describes the components, structure and programming of a microprocessor

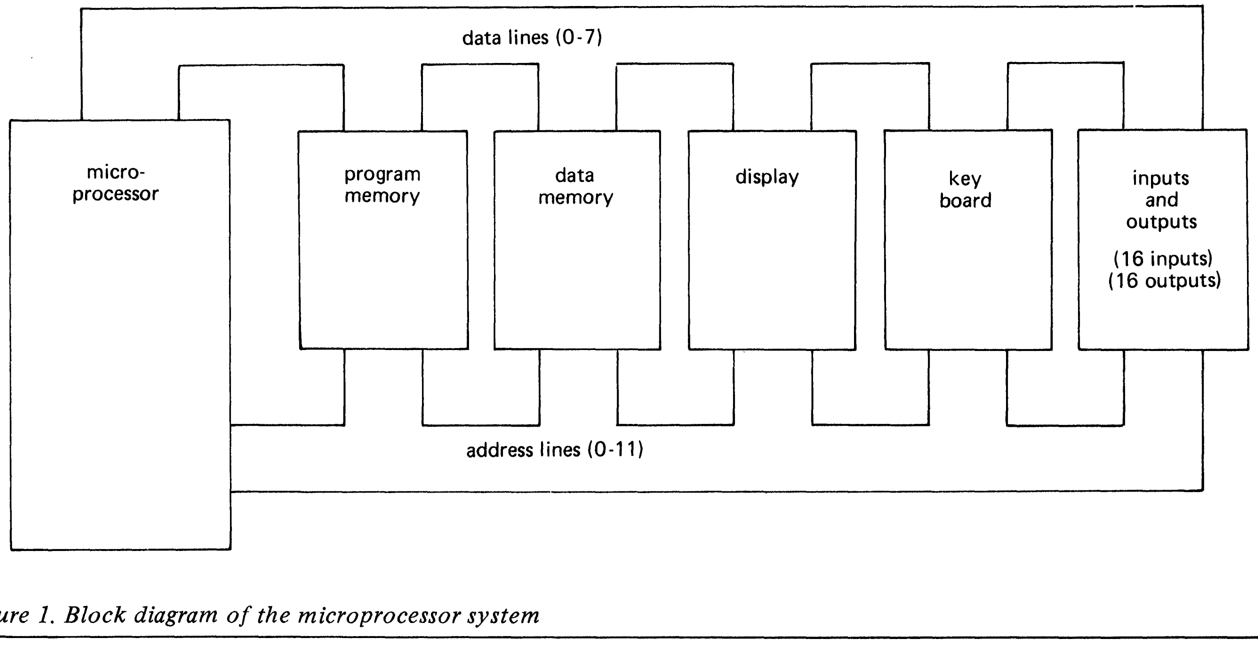

*Figure 1. Block diagram of the microprocessor system*

based controller constructed within the laboratory, for use in the development of automatic systems. The description is in two parts (a) the microprocessor components and (b) the program.

The controller has been designed to:

(1) Accept and store a sequential list of instructions in the order in which they must be carried out.
(2) Once initiated, carry out the sequence of instructions with no further intervention by the operator and to stop at the end of the list.
(3) Repeat a sequence until intervention by the operator causes it to stop.

### The microprocessor system

While most other 8 bit processors would have been suitable, a National Semiconductor SC/MP microprocessor was used for the system which is shown in block form in Figure 1. The SC/MP is an 8 bit microprocessor with an 8 bit data bus and a 12 bit address bus. It has an addressing capability of 65,536 words and will interface directly to a range of suitable peripherals such as teletypes and printers. A general purpose instruction set is provided which will perform either 8 bit binary or 2 digit binary coded decimal (BCD) arithmetic. The program memory, used to store the operating system, is a National Semiconductor MM 5204Q programmable read only memory (PROM) with 512 words of storage. The data memory is formed from two National Semiconductor 2112–N2 random access memories (RAM) giving 256 words of storage; this is used to store the users control sequence. Both memories are connected to the 8 bit data lines and are internally buffered to allow them to be switched in or out of use. The particular memory location is selected by an address value placed on the 12 address lines. The display, which is formed from four pairs of seven segment gas discharge displays which show two decimal digits each, also uses the data and address lines. The information is latched into the display to provide a coherent, readable code. The information which can be displayed in conjunction with the keyboard is:

(a) The current step number during instruction input.
(b) The current step number during normal running phase.
(c) The next step number during instruction input.
(d) The code of the current step number during instruction input.

A block diagram on one pair of displays and latches is shown in Figure 2; the whole display consists of four of these circuits.

The keyboard is also connected onto the date lines as an input to the microprocessor. The keys consist of four pairs of decimal thumbwheel switches and four pushbutton switches. The thumbwheel switches are used for setting up the required control code and are set out to correspond to the display format. The four pushbutton switches consist of:

(a) A 'RUN' switch changes the microprocessor from parameter 'set up' entry mode into operational mode.
(b) The 'RESET' switch is used to initialise the microprocessor into the parameter entry mode.
(c) The 'LOAD' switch is used to enter the parameters into memory; whilst pressed the current instruction number is displayed and when released the values present on the thumbwheel switches are entered into memory.
(d) The 'INCREMENT MEMORY' switch is used to step onto the next instruction location. When held 'on', the next instruction number is displayed and when released the code of this instruction is displayed.

Figure 3 shows a block diagram of one pair of thumbwheel switches and the pushbuttons. Each switch except the RESET and RUN is selected by an enable signal which connects the switch onto the data lines via a buffer circuit. The RESET switch is connected straight to the reset line of the microprocessor and the RUN switch is used to inhibit the interrupt system.

The input/output system is also connected onto the data lines and each individual input or output is selected by means of an address. The inputs to the microprocessor are isolated by opto-isolators and the outputs from the microprocessor drive relays. This gives complete isolation from the external circuitry. The inputs are read by the microprocessor when required and the outputs are set into the required state by the microprocessor and remain in this state until changed by the microprocessor.

Figures 4 and 5 show block diagrams of the input and output systems. The relay outputs have a capability for switching up to 240 volts at 2 amps. In addition to the circuits described above, there is an internal clock which provides times from 1 second to 99 minutes 59 seconds. This clock is used for measuring the delay times which have been set by the operator.

### The program

The program system is organised in two parts.

(a) The program which accepts the information regarding the control of the automatic system.

(b) The program which actually controls the automatic system using the information obtained from part (a)

(a) This first part of the program is brought into operation when the unit is switched on, or when the RESET button is pushed. The function of the program is to read in the coded instructions which have been set up on the thumbwheel switches by the operator, display the instruction and its position in the sequence of instructions. The operation of the program is controlled by the keyboard switches, described earlier, which are monitored until one is activated. The program then performs the task related to that switch before

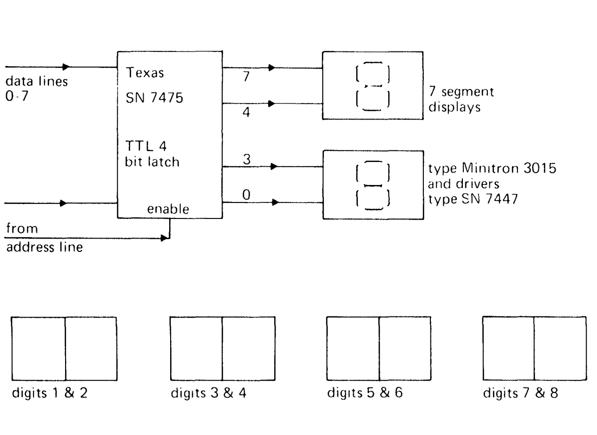

*Figure 2. Block diagram of display and latch.*

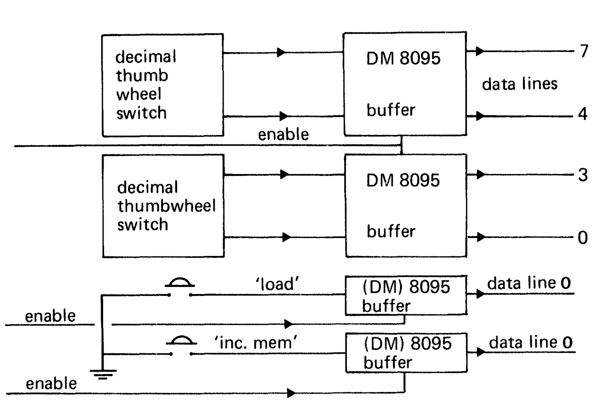

*Figure 3. Block diagram of thumbwheel and pushbutton switches.*

returning to its monitoring role. The program remains in this mode until the RUN switch is operated, at which point the second part of the program comes into operation.

(b) The second part of the program is brought into operation by operating the run switch. The first control instruction in the sequence is selected and the specified input number is checked. The program then waits until the input comes on and the specified output is then turned on or off in accordance with the control instruction. Where an instruction does not specify an input number, then a time is included in that instruction. This time is the delay between the execution of the last instruction and the execution of the current instruction. To execute the time delay, the internal timer is reset and then allowed to count in 1 second steps. At each step the time value is compared to the value specified in the control instruction. When the two values coincide the appropriate output action is taken. The program steps sequentially through each control instruction until the last one which must be one of two fixed alternatives (1) STOP or (2) RETURN TO FIRST INSTRUCTIONS AND CONTINUE. The latter provides for continuous looping round the sequence.

To illustrate the manner in which the controller is set up to deal with a specific problem, a simple four position probe can be considered. The probe has two vertical positions ie, 'raised' and 'lowered' and two horizontal positions 'sample' and 'wash'. The sequence of events would be to raise the probe from the wash position, rotate to the sample position and lower into the sample, and then reverse the sequence.The control is exercised using two outputs from the controller which may be used to drive electro-pneumatic actuators. Output 1 controls the rotation of the probe, Output 2 controls the vertical movement of the probe.

**Table 1. Program**

| Step No. | Instruction | O/P | Effect |
|---|---|---|---|
| 1 | 00 00 05 02 | OFF | after 5s raise probe |
| 2 | 00 00 02 01 | ON | after 2s rotate probe |
| 3 | 00 00 02 02 | ON | after 2s lower probe |
| 4 | 00 00 05 02 | OFF | after 5s raise probe |
| 5 | 00 00 01 01 | OFF | after 1s rotate probe |
| 6 | 00 00 02 02 | ON | after 2s lower probe |
| 7 | 99 xx xx xx | x | return to step 1 |

(x indicates instruction values are not important)

### Discussion

The unit, which can be seen in Figure 6, is quite compact and has been in general use for several months. Operators are finding it easier and quicker to set up and use than the equivalent electro-mechanical system. The unit has been used for developing part of an automatic quinizarin detector, as well as more general applications. The users are keeping note of any extra features that could usefully be added to any future unit. Once the final system configuration has been obtained for the automatic system, then either a hard wired logic system or a dedicated microprocessor system is built to perform the task, the method chosen depending on system size and complexity.

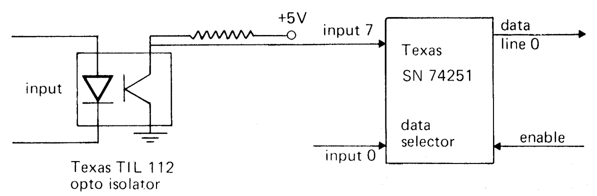

*Figure 4. Block diagram of input system.*

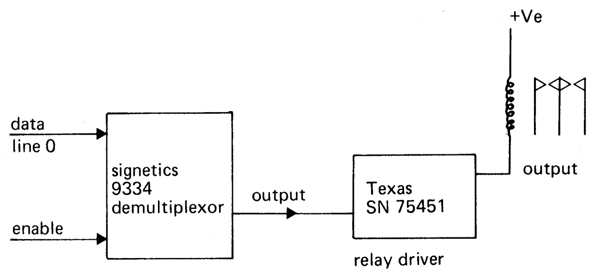

*Figure 5. Block diagram of output system.*

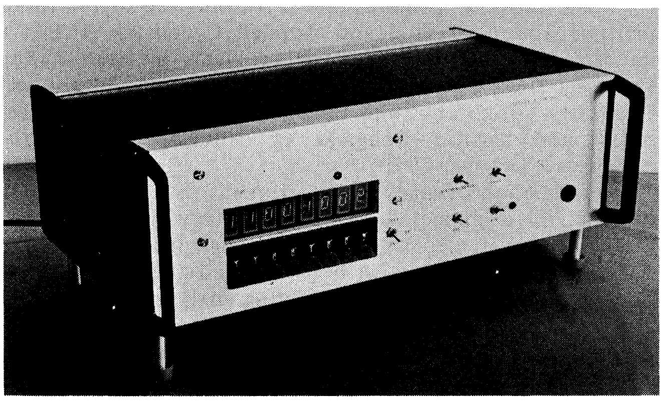

*Figure 6. Photograph of unit.*

REFERENCES

[1] Morley, F., O'Neill, I.K., Pringuer, M.A., Stockwell, P.B., *Analytical Chemistry* 1979, **51**, 579.

[2] Morley, F. and Stockwell, P.B., *Journal of Dentistry* 1977, **5**, (1), 39–41.

[3] Bunting, W., Morley, F., Telford, I., Stockwell, P.B., *The Analyst*, 1975, **100**, 359.

[4] Dennis, A.L., Jackson, C.J., Porter, D.G. Stockwell, P.B., *The Analyst*, 1978, **103**, 317–331.

[5] Bunting and Stockwell, P.B., *The Analyst*, 1978, **103**, (1222), 72–78.

ACKNOWLEDGEMENTS

The author would like to thank Mr. R. Wardale for the construction of the system, and the Goverment Chemist for permission to publish this paper.

